# Prospective Trial of Radiofrequency Catheter Ablation of High Dominant Frequencies After Pulmonary Vein Isolation in Non‐Paroxysmal Atrial Fibrillation (PAD‐AF Trial): A Multicenter, Randomized Clinical Trial

**DOI:** 10.1111/jce.70357

**Published:** 2026-05-03

**Authors:** Koji Kumagai, Tomoaki Hasegawa, Daisuke Kutsuzawa, Yuichi Hanaki, Masahide Harada, Hitoshi Hachiya, Yuhi Hasebe, Kazuki Noda, Minoru Yambe, Hirohito Metoki, Kazutaka Aonuma

**Affiliations:** ^1^ Department of Cardiovascular Medicine Tohoku Medical and Pharmaceutical University Miyagi Japan; ^2^ Division of Cardiology, Mito Saiseikai General Hospital Ibaraki Japan; ^3^ Division of Cardiology Yamagata University Hospital Yamagata Japan; ^4^ Division of Cardiology, University of Tsukuba Hospital Ibaraki Japan; ^5^ Division of Cardiology Fujita Health University Aichi Japan; ^6^ Division of Cardiology, Tsuchiura Kyodo General Hospital Ibaraki Japan; ^7^ Division of Cardiology, Sendai City Medical Center Sendai Open Hospital Sendai Japan; ^8^ Division of Public Health, Hygiene, and Epidemiology Tohoku Medical and Pharmaceutical University Miyagi Japan

**Keywords:** atrial fibrillation, catheter ablation, dominant frequencies, pulmonary vein isolation

## Abstract

**Background:**

The role of dominant frequency (DF)–guided ablation after pulmonary vein isolation (PVI) in persistent atrial fibrillation (AF) remains uncertain. We evaluated the clinical and mechanistic impact of DF mapping in a multicenter randomized study.

**Methods and Results:**

In this multicenter, prospective study, 103 patients were enrolled. Patients with high‐DF sites (≥ 7 Hz) were randomized 1:1 to PVI plus DF ablation (DF group, *n* = 32) or PVI alone (PVI group, *n* = 32). Patients without high‐DF sites (< 7 Hz, *n* = 39) underwent PVI only (non‐DF group; exploratory cohort). The primary endpoint was freedom from documented AF recurrence without antiarrhythmic drugs (AADs) at 12 months. AF‐free survival off AADs was 81.3% in the DF group versus 68.9% in the PVI group (*p* = 0.228) at 12 months. Arrhythmia‐free survival with or without AADs was 78.1% versus 65.6% (*p* = 0.263). The non‐DF group showed the most favorable outcomes (83.3%, *p* = 0.065 vs PVI group). No adverse events were associated with DF mapping or ablation. Multivariate analysis identified right atrial (RA) low‐voltage area extent (HR 1.031, 95% CI 1.005–1.058, *p* = 0.018) and LA diameter (HR 0.899, 95% CI 0.816–0.991, *p* = 0.032) as independent predictors of recurrence.

**Conclusions:**

In this multicenter randomized trial, adjunctive DF‐guided ablation following PVI did not result in a statistically significant improvement in arrhythmia‐free survival compared with PVI alone. However, the absence of high‐DF sites was associated with favorable outcomes, and RA low‐voltage burden emerged as an independent predictor of recurrence, supporting the potential mechanistic value of DF mapping and highlighting the prognostic importance of right atrial structural remodeling.

**Trial Registration:**

UMIN000042543.

## Graphical abstract

In persistent AF, adjunctive dominant frequency‐guided ablation after pulmonary vein isolation did not significantly improve outcomes. Limited overlap between high‐frequency sites and low‐voltage areas suggests functional–structural dissociation, while right atrial low‐voltage burden predicts recurrence.

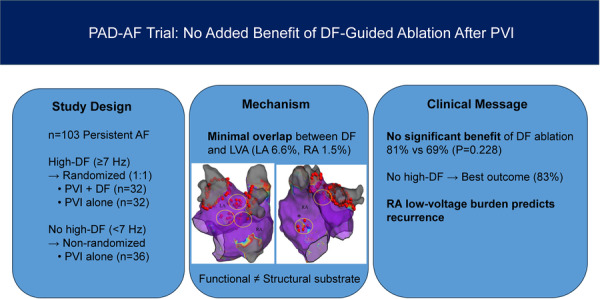

**Abbreviations**
AF: atrial fibrillation
DFs: dominant frequencies
PVI: pulmonary vein isolation

## 1 Introduction

Pulmonary vein isolation (PVI) is the cornerstone of catheter ablation for atrial fibrillation (AF) [1], but outcomes in persistent AF remain suboptimal despite numerous adjunctive strategies targeting non‐pulmonary vein substrates. Various approaches—including linear ablation, complex fractionated atrial electrogram (CFAE) ablation, low‐voltage area (LVA)‐guided substrate modification, and rotor elimination—have been tested [2, 3, 4, 5, 6], yet randomized trials have largely failed to show consistent benefit over PVI alone [7, 8, 9, 10].

Spectral analysis with dominant frequency (DF) mapping has been proposed to identify atrial regions harboring high‐frequency activity, which may represent AF‐maintaining substrates [11, 12]. Experimental and clinical studies have demonstrated that DF‐guided ablation can prolong AF cycle length or even terminate AF in selected cases [11, 12]. However, the reproducibility and clinical relevance of DF sites remain uncertain, in part due to temporal instability and limited correlation with stable driver regions [13, 14].

Importantly, the spatial relationship between DF sites and structural remodeling such as LVA has not been fully clarified [15, 16, 17, 18, 19]. Previous observational studies have suggested that only a subset of high‐DF regions overlap with diseased atrial tissue [16, 17, 18], raising the question of whether DF mapping provides independent mechanistic information.

The present prospective, multicenter study (PAD‐AF trial) was designed to evaluate the role of DF mapping in patients with persistent AF. Specifically, we aimed to determine whether adjunctive DF ablation after PVI improves arrhythmia outcomes, and to explore the mechanistic significance of DF sites in relation to LVA and clinical recurrence.

## 2 Methods

### 2.1 Study Design

The PAD‐AF (Prospective trial of Radiofrequency Catheter Ablation of High Dominant Frequencies after PVI in non‐paroxysmal atrial fibrillation) trial was an investigator‐initiated, multicenter study consisting of a randomized comparison and an exploratory observational cohort. The trial was conducted at six centers in Japan between September 2020 and December 2023. The protocol for this research project was approved by a suitably constituted Ethics Committee of Tohoku Medical and Pharmaceutical University (Date of IRB approval: May 18, 2019; Approval number, 2019‐2‐115) and it conformed to the provisions of the Declaration of Helsinki. The trial was approved by the ethics committee at each center before enrollment of any patients. Written informed consent was obtained from all participants. The trial was registered and partially supported by a grant‐in‐aid for scientific research from the Japanese Ministry of Education, Culture, Sports, Science, and Technology (No. 20K08495).

### 2.2 Study Population

Patients with symptomatic persistent (≥ 7 days and < 1 year) or long‐standing persistent AF (≥ 1 year) were eligible for inclusion [1]. Key exclusion criteria included: prior AF ablation; significant structural heart disease; left atrial diameter > 50 mm; left ventricular ejection fraction < 50%; chronic kidney disease (eGFR < 50 mL/min/1.73 m²); active thyroid dysfunction; pregnancy; life expectancy < 1 year; recent cardiovascular events or bleeding requiring hospitalization; and inability to maintain anticoagulation therapy. Antiarrhythmic drugs (AADs) were discontinued at least five half‐lives before the procedure, and none of the patients received amiodarone.

During the study period, patients with persistent AF undergoing catheter ablation at the participating centers were prospectively screened for study eligibility according to predefined inclusion and exclusion criteria. However, not all patients undergoing catheter ablation for non‐paroxysmal AF during the study period were systematically screened for study participation, as screening depended on the availability of research personnel and institutional workflows at each center. Eligible patients were enrolled consecutively at each participating center. Among those who met the inclusion criteria, patients who demonstrated persistent high dominant‐frequency sites following PVI were eligible for randomization.

After PVI and DF mapping, patients were categorized according to the presence of high‐dominant frequency (DF) sites (≥ 7 Hz). Those with high‐DF sites were randomized 1:1 to adjunctive DF ablation plus PVI (DF group) or PVI alone (PVI group). Patients without high‐DF sites (< 7 Hz) underwent PVI only and were analyzed as a non‐randomized exploratory cohort (non‐DF group).

### 2.3 Ablation Procedure

All procedures were performed under conscious sedation and uninterrupted oral anticoagulation. After single transseptal access under intracardiac echocardiographic guidance, a single bolus of 5000 U of heparin was administered. Additional heparin boluses were administered as needed to maintain an activated clotting time > 300 s throughout the procedure. The 3D LA geometry was created using a 7‐F decapolar circular catheter (EPstar Libero, Japan Lifeline Co. Ltd.). The whole LA was divided into eight areas (PVs, roof, left atrial appendage [LAA], septum, and lateral, anterior, inferior, and posterior regions) for a location analysis of the LVA and DFs [13, 14]. The mapping points in each region were similar in number and nearly equally distributed. PVI was performed using a 3.5‐mm irrigated‐tip radiofrequency catheter (FlexAbility, Abbott), delivering point‐by‐point ablation with each radiofrequency energy application of 40 s at guided by a NavX system (Abbott) as described previously [15, 16, 17, 18]. The temperature and power were limited to 42°C and 35 W (25 W near the esophagus), with irrigation at 13 mL/min. Bidirectional PV isolation was confirmed in all cases. During ongoing AF, the entrance block was confirmed by elimination of PV potentials; exit block was assessed after cardioversion to sinus rhythm with differential pacing. When AF continued after the PVI, DF mapping was performed using an Advisor HD Grid during AF. Finally, a LA voltage map was performed using the Advisor HD Grid, during pacing from the distal CS after external cardioversion. At the time of study initiation (2020–2023), radiofrequency energy was the standard‐of‐care ablation modality across all participating centers; therefore, RF was uniformly applied to minimize procedural variability.

Once in SR, decremental pacing (10 ms steps from 250 to 200 ms, over a period of 10 s) at an output of 10 mA and 2 ms pulse width was performed from the distal CS once, in an attempt to induce an atrial tachyarrhythmia without an isoproterenol injection. An induced AF/AT (atrial tachyarrhythmias) was defined as one sustained for at least 2 min [1]. When AF/AT continued, external cardioversion was performed. When cavotricuspid isthmus (CTI)‐dependent AFL was induced, a CTI ablation was performed.

### 2.4 DF Mapping and Ablation

If AF persisted after PVI, bi‐atrial DF mapping was performed during ongoing AF using the Advisor™ HD Grid catheter (Abbott). A mean number of mapping points per patient was acquired across both atria (LA: 11686 ± 7121; RA: 9181 ± 4986). Mapping points were acquired with a similar distribution across the predefined atrial regions to ensure balanced regional assessment. As described previously [15, 16, 17, 18], recordings from each atrial region were analyzed using fast Fourier transform (FFT) over 5‐second epochs. Signals were truncated to 5 s at a sampling rate of 1000 Hz, providing 4096 points for analysis (resolution 0.50 Hz). The signals were rectified and processed by a Hanning window function and filtered from 2 to 20 Hz. The DF analysis was performed by an offline FFT analysis using the software implemented in the polygraph (RMC‐5000; Nihon Kohden Co.) in real time and then the DF values were input manually into the NavX system. The DF was defined as the frequency with the maximum spectral power, and only sites with a regularity index ≥ 0.2 were included. High‐DF sites were defined as those ≥ 7 Hz. High‐DF was prespecified as ≥ 7.0 Hz with RI ≥ 0.2 based on prior human AF spectral studies and our institutional series, in which ≥ 7 Hz captured the upper‐tail of AF activation frequency with acceptable reproducibility [13, 14].

All high‐DF sites were targeted for ablation in descending order of frequency for 40–60 s until local electrograms within the surrounding area of the center of the high‐DF site were abolished or significantly attenuated, because the characteristics of rotor drift and temporal instability may make it difficult to map rotors completely using the Advisor HD Grid [14]. If AF persisted after DF ablation, external cardioversion was performed. Patients without any high‐DF sites underwent PVI only.

### 2.5 Low‐Voltage Mapping

After the PVI, DF mapping, and ablation, a detailed bipolar LA and RA voltage map was constructed during CS pacing after external cardioversion. The LVA mapping method has been described previously [4]. The mapping points were systematically acquired with an Advisor HD Grid ^TM^. An interpolation threshold of 10 mm on the NavX system was used for the surface color projection. Adequate endocardial contact was evaluated by stable electrograms and consideration of the distance to the geometry surface. Bipolar electrograms were filtered by a bandpass to frequencies between 30 and 500 Hz. In accordance with the previous studies [4], an LVA was defined as an area with a bipolar peak‐to‐peak electrogram amplitude of < 0.5 mV and electrical scar areas as < 0.1 mV and covering > 5% of the LA body surface area. The LA surface area was defined as the LA body area without the PV antrum regions inside the PVI line. A fusion of multidetector computed tomography (MDCT) imaging and electroanatomical mapping was performed. The spatial overlap analysis, but not DF identification itself, was assessed manually by 2 independent blinded observers.

### 2.6 Postprocedural Management and Follow‐up

AADs were not prescribed beyond the 3‐month blanking period unless clinically indicated. Follow‐up visits were scheduled at 1‐, 3‐, 6‐, and 12‐months post‐ablation. Evaluation included clinical interview, ECG, blood tests, at 1‐, 3‐, 6‐, and 12‐months, and 24‐h Holter monitoring at 12 months. Additional event monitoring was performed when symptoms occurred. Arrhythmia recurrence was defined as any documented AF episode > 30 s beyond the blanking period [1].

### 2.7 Endpoints

The primary endpoint was freedom from recurrent AF lasting > 30 s at 12 months without the use of AADs after a single procedure to isolate AF‐specific effects. Secondary endpoints included overall atrial arrhythmia recurrence (AF/AFL/AT) with or without AADs, procedural characteristics, and safety outcomes. Analyses including the non‐DF group were considered exploratory.

### 2.8 Statistical Analysis

Continuous variables are presented as mean ± standard deviation or median (IQR; interquartile range), and categorical variables as counts and percentages. Between‐group comparisons were performed using the Student's *t*‐test or Mann–Whitney U test for continuous variables, and Fisher's exact test for categorical variables. The randomized comparison between DF and PVI groups represented the primary analysis. Analyses involving the non‐DF cohort were exploratory. Predictors of arrhythmia recurrence were assessed using univariate and multivariate Cox proportional hazards regression models. Variables with *p* < 0.1 in the univariate analysis were included in the multivariate analysis. Kaplan–Meier analysis with log‐rank testing was used to compare arrhythmia‐free survival. A two‐sided *p* value < 0.05 was considered statistically significant. All statistical analyses were performed using SPSS for Windows version 27.0 (IBM SPSS, Armonk, NY: IBM Corp.).

The PAD‐AF trial was designed as an exploratory mechanistic randomized study evaluating the potential clinical impact of adjunctive dominant‐frequency–guided ablation following PVI. Because of the limited prior data available regarding DF‐guided ablation in persistent atrial fibrillation, a formal power calculation was not feasible at the time of study design.

## 3 Results

### 3.1 Study Population

**Figure 1:**
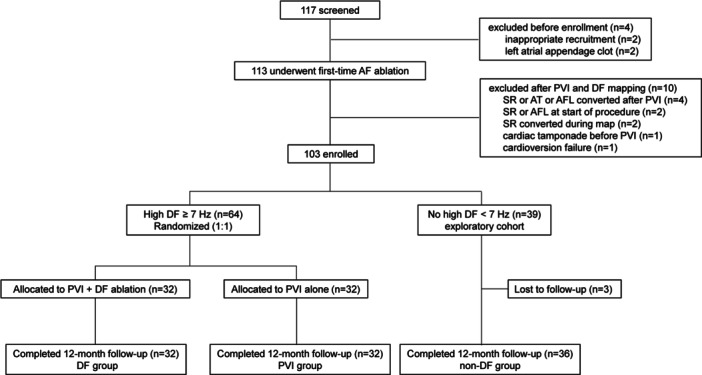
Patient flow, randomization, and analysis population in the PAD‐AF trial. Of 117 patients screened, 103 were enrolled: 64 with high‐DF sites were randomized to DF (*n* = 32) or PVI (*n* = 32) groups, and 36 without high‐DF sites formed a non‐DF cohort.

Between September 2020 and December 2023, 117 patients were screened, and 103 were enrolled. Among them, 64 patients with high‐DF sites (≥ 7 Hz) persisting after PVI were randomized to either the DF group (PVI + DF ablation, *n* = 32) or the PVI group (PVI alone, *n* = 32). Thirty‐nine patients without high‐DF sites (< 7 Hz) comprised an exploratory observational cohort (non‐DF group). Figure 1 shows the Patient flow, randomization, and analysis population in the PAD‐AF trial.

Baseline characteristics are summarized in Table 1. Baseline characteristics were comparable between DF and PVI groups, whereas the non‐DF group had a lower proportion of males and higher CHADS₂ scores.

**Table 1:** Patient characteristics.

	DF group (*N* = 32)	PVI group (*N* = 32)	Non‐DF group (*n* = 36)	*p* value*
Age, y	62 ± 8.5	65 ± 9.1	68 ± 7.5	0.255
Male, *n* (%)	25 (78)	30 (94)	26 (74)	0.148
BMI, kg/m²	26 ± 3.8	25 ± 3.2	26 ± 4.0	0.220
Persistent AF, *n* (%)	20 (63)	15 (47)	15 (42)	0.315
Longstanding persistent AF, *n* (%)	12 (37)	17 (53)	21 (58)	0.315
Duration of AF, median (IQR), mo	9 (6–22)	12 (7–33)	12 (7–23)	0.293
LA diameter (mm)	43 ± 5.2	43 ± 4.5	44 ± 3.3	0.777
LVEF (%)	61 ± 7.8	60 ± 6.7	59 ± 7.5	0.621
Congestive heart failure, *n* (%)	3 (9)	3 (9)	1 (3)	1.000
Hypertension, *n* (%)	19 (59)	17 (53)	23 (37)	0.801
Diabetes mellitus, *n* (%)	6 (19)	4 (13)	9 (25)	0.732
Prior stroke or TIA, *n* (%)	0 (0)	1 (3)	4 (11.1)	1.000
CHADS2 score	0.9 ± 0.7	0.8 ± 0.9	1.3 ± 0.9	0.873
BNP (pg/mL)	117 ± 102	117 ± 59	151 ± 75	0.987
eGFR (mL/min/1.73 m²)	68 ± 13	66 ± 11	67 ± 15	0.346
Baseline AADs				
Class I, *n*	1	1	2	1.000
Class III, *n*	1	0	1	0.313

Abbreviations: AF = atrial fibrillation, AADs = antiarrhythmic drugs, BNP = B‐type Natriuretic Peptide; BMI = body mass index, DF = dominant frequency, GFR = glomerular filtration rate, LA = left atrium, LVEF = left ventricular ejection fraction, PVI = pulmonary vein isolation, RA = right atrium.

* *p* values are shown for comparison between the randomized groups (DF vs. PVI). The non‐DF group is presented for descriptive purposes only.

### 3.2 Procedural Characteristics

All pulmonary veins were successfully isolated. DF ablation did not acutely terminate AF in any case. Procedure time, fluoroscopy time, and RF duration were similar between DF and PVI groups (Table 2). Cavotricuspid isthmus ablation was performed when typical atrial flutter was inducible. No adverse events, including pericardial tamponade, stroke, pulmonary vein stenosis, or atrial‐esophageal fistula, were associated with DF mapping or ablation.

**Table 2:** Acute procedural data.

	DF group (*N* = 32)	PVI group (*N* = 32)	Non‐DF group (*n* = 36)	*p* value*
Electrophysiological characteristics				
Max DF in LA, Hz	7.7 ± 0.5	7.5 ± 0.5	—	0.193
RI of max DF in LA	0.3 ± 0.1	0.3 ± 0.1	—	0.113
Max DF in RA, Hz	7.6 ± 0.5	7.5 ± 0.5	—	0.570
RI of max DF in RA	0.3 ± 0.1	0.4 ± 0.4	—	0.236
High‐DF sites in LA, median (IQR), *n*	5.5 (2.3–7.8)	3.5 (1.0–9.0)	—	0.559
High‐DF sites in RA, median (IQR), *n*	2.0 (0–4.8)	1.5 (0–3.8)	—	0.566
LVA/LA surface area after PVI, median (IQR), %	1.9 (0–4.7)	1.3 (0.4–4.6)	1.8 (0.5–5.5)	0.219
LVA/RA surface area after PVI, median (IQR), %	3.6 (1.5–7.4)	3.5 (1.6–7.2)	4.2 (0.7–6.7)	0.142
Patients with High‐DF sites in LA overlapped with LVA, *n* (%)	3 (9)	6 (19)	—	0.476
Patients with High‐DF sites in RA overlapped with LVA, *n* (%)	1 (3)	2 (6)	—	1.000
Procedural parameters				
Cavotricuspid isthmus line, *n* (%)	5 (16)	9 (27)	6 (20)	0.226
Inducibility of AF, *n* (%)	1 (3)	1 (3)	4 (15)	0.982
Inducibility of AFL/AT, *n* (%)	4 (13)	8 (26)	5 (19)	0.179
Total procedure time, min	194 ± 58	178 ± 56	164 ± 27	0.286
RF time for PVI, min	27 ± 14	27 ± 16	27 ± 19	0.892
DF ablation time, min	10 ± 9	—	—	—
DF mapping time, min	30 ± 19	30 ± 23	—	0.962
Fluoroscopy time, min	61 ± 34	50 ± 33	50 ± 21	0.208
Fluoroscopy exposure, mGy	455 ± 363	375 ± 370	336 ± 230	0.385
Cardiac tamponade, *n*	0	0	0	1.000
Stroke, *n*	0	0	0	1.000
Antiarrhythmic drugs during 1 year follow‐up, *n* (%)	3 (9)	6 (19)	3 (8)	0.213

Abbreviations: AF = atrial fibrillation, AADs = antiarrhythmic drugs, DF = dominant frequency, LA = left atrium, LVEF = left ventricular ejection fraction, PVI = pulmonary vein isolation, RA = right atrium, RI = regularity index.

* *p* values represent comparisons between the randomized groups (DF vs. PVI). The non‐DF group is presented for descriptive purposes only.

### 3.3 Dominant Frequency Mapping and LVA Analysis

**Figure 2:**
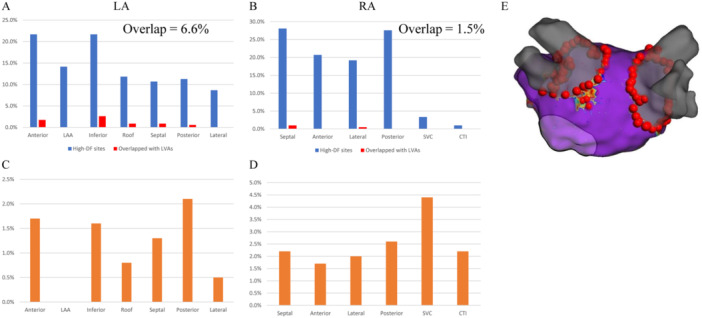
Spatial distribution of high dominant frequency (DF) sites and low‐voltage area (LVA) in the left and right atria. The spatial overlap between high‐DF sites and LVA was limited (6.6% in the LA and 1.5% in the RA), indicating spatial dissociation between functional (frequency‐based) and structural (voltage‐defined) substrates. (A, B) Distribution of high‐DF sites in the left atrium (LA) and right atrium (RA), respectively (a total of 346 sites in LA and 203 in RA). High‐DF sites were predominantly located in the anterior, inferior, and left atrial appendage regions in the LA, and in the septal, posterior, and anterior walls in the RA. Overlapping regions with LVA are indicated in red. (C, D) Distribution of LVA (< 0.5 mV) in the LA and RA, expressed as the mean extent of LVA in each anatomical region, respectively. LVA was predominantly observed in the posterior, anterior, and inferior walls in the LA, and in the superior vena cava (SVC), posterior, and septal walls in the RA. The spatial distribution of LVA differed from that of high‐DF sites. (E) Representative voltage map of the LA demonstrating minimal spatial overlap between high‐DF sites and LVA.

The maximum DF values in the LA and RA were similar between the DF and PVI groups (LA: 7.7 ± 0.5 vs. 7.5 ± 0.5 Hz, *p* = 0.193; RA: 7.6 ± 0.5 vs. 7.5 ± 0.5 Hz, *p* = 0.570) (Table 2). The number of high‐DF sites identified in the LA and RA did not differ significantly (LA: 5.5 [IQR 2.3–7.8] vs. 3.5 [IQR 1.0–9.0], *p* = 0.559; RA: 2.0 [IQR 0–4.8] vs. 1.5 [IQR 0–3.8], *p* = 0.566). The spatial distribution of high‐DF sites in the LA predominantly included the anterior, inferior, and LAA; in the RA, high‐DF sites were commonly found on the septal, posterior, and anterior wall (Figure 2A,B).

The regional distribution of LVA, expressed as mean extent in each anatomical region, was predominantly observed in the posterior, anterior, and inferior walls; in the RA, LVA was commonly found in the SVC, posterior, and septal walls (Figure 2C,D). The extent of LVA (< 0.5 mV) did not differ between groups. (LA: 1.9 [IQR 0–4.7] % vs. 1.3 [IQR 0.4–4.6] %, *p* = 0.219; RA: 3.6 [IQR 1.5–7.4] % vs. 3.5 [IQR 1.6–7.2] %, *p* = 0.142).

**Figure 3:**
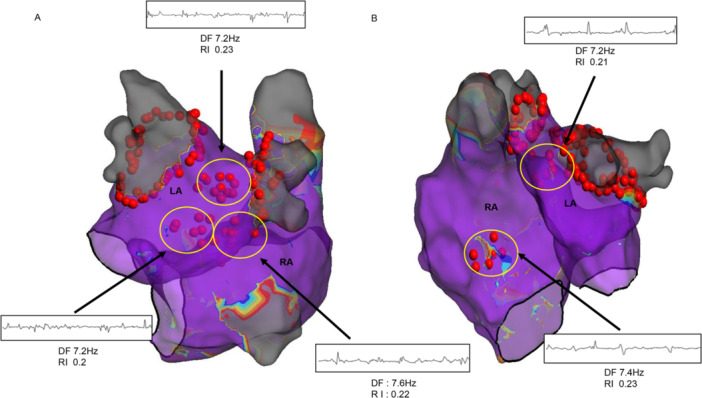
Voltage mapping and ablation lesion sets at high dominant frequency (DF) sites in (A) Voltage map of the left atrium (LA). (B) Voltage map of the right atrium (RA).Voltage mapswith low‐voltage areas (LVA) and ablation lesion sets at high‐DF sites in the LAand RA are shown. Even in patients with minimal structural remodeling (LVA/LAsurface area: 3.4%), high‐DF sites were distributed across multiple atrialregions, including the roof, posterior, and inferior walls in the LA, and theanterior wall in the RA. High‐DF sites were located both within and outsideLVAs, demonstrating limited spatial concordance between functional andstructural substrates. Importantly, high‐DF sites were also observed in the RA,consistent with biatrial substrate involvement. Red tags indicate ablationpoints at the pulmonary vein isolation sites and at high‐DF regions. Colorcoding is defined as follows: < 0.1 mV = scar (gray), 0.1–0.5 mV = diseasedatrial tissue, and > 0.5 mV = healthy atrial myocardium (purple). DF = dominant frequency; RI = regularity index. Left panel: posterior‐anterior view; right panel: LAO superior view.

Limited overlap between high‐DF sites and LVA in the LA and RA was observed in Figure 2. The overall overlap rate was 6.6% in LA and 1.5% in RA, suggesting spatial dissociation between functional and structural substrates. Representative voltage maps showing LVA and ablation at high‐DF sites in the LA and RA are presented in Figure 3.

### 3.4 Primary and Secondary Outcomes (Randomized Comparison)

**Figure 4:**
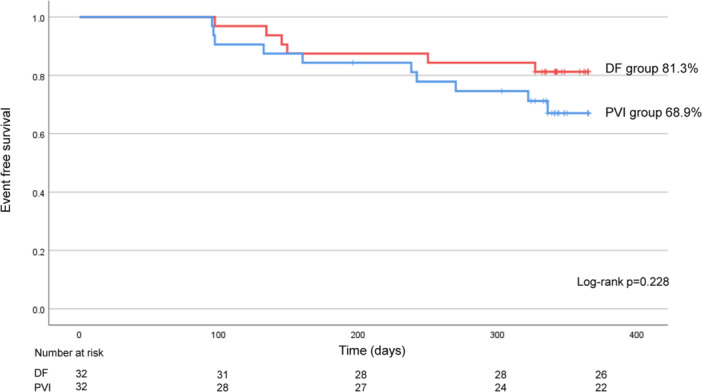
Kaplan–Meier analysis of event‐free survival showing cumulative freedom from AF recurrence off AADs in the DF and PVI groups. At the 12‐month follow‐up, freedom from recurrent AF without AADs after a single procedure was achieved in 81.3% of patients in the DF group compared to 68.9% in the PVI group (*p* = 0.228 by log‐rank test).

**Figure 5:**
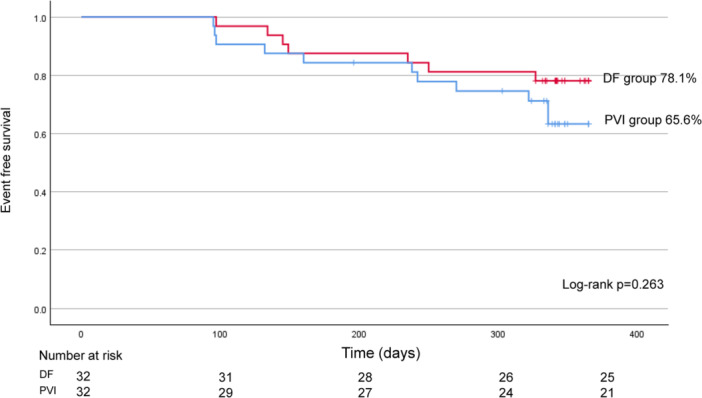
Kaplan–Meier analysis of event‐free survival showing cumulative freedom from AF/AFL/AT recurrence on or off AADs in the DF and PVI groups. At the 12‐month follow‐up, freedom from recurrent AF, AFL, or AT with or without AADs after a single procedure was achieved in 78.1% of patients in the DF group compared to 65.6% in the PVI group (*p* = 0.263 by log‐rank test).

At 12 months, freedom from AF recurrence without AADs after a single procedure (primary endpoint) was achieved in 81.3% of patients in the DF group and 68.9% in the PVI group (log‐rank *p* = 0.228) (Figure 4). Freedom from any atrial arrhythmia (AF/AFL/AT) with or without AADs (secondary endpoint) was 78.1% versus 65.6% (*p* = 0.263) (Figure 5). These differences did not reach statistical significance, although numerical trends favored adjunctive DF ablation.

### 3.5 Exploratory Analysis (Non‐Randomized Cohort)

**Figure 6:**
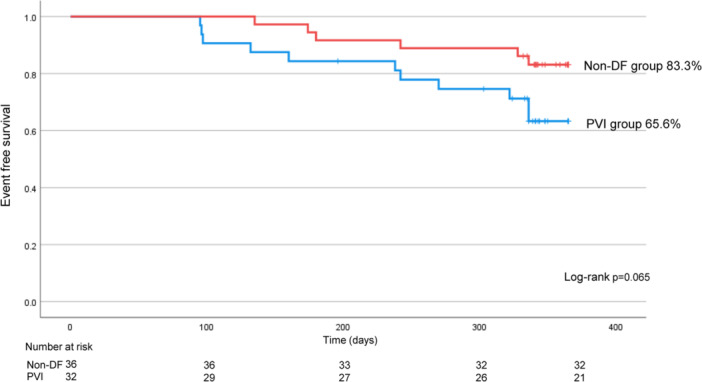
Kaplan–Meier analysis of event‐free survival showing cumulative freedom from AF/AFL/AT recurrence on or off AADs in the non‐DF and PVI groups. At the 12‐month follow‐up, the non‐DF group showed an arrhythmia‐free rate of 83.3% with or without AADs, which trended higher compared to 65.6% in the PVI group (*p* = 0.065 by log‐rank test).

In the non‐DF group, atrial arrhythmia‐free survival with or without AADs at 12 months was 83.3%, which appeared higher than the PVI group (65.6%, *p* = 0.065). As this group was not randomized, these findings should be interpreted as exploratory and hypothesis‐generating (Figure 6).

### 3.6 Predictors of Atral Arrhythmia Recurrence

In univariate Cox regression analysis in the patients with high‐DF sites, LA diameter (HR 0.911, 95% CI 0.833–0.996, *p* = 0.040) and extent of RA LVA (HR 1.030, 95% CI 1.003–1.057, *p* = 0.028) were associated with atrial arrhythmia recurrence with or without AADs. Multivariate analysis identified the extent of RA LVA (HR 1.031, 95% CI 1.005–1.058, *p* = 0.018) and LA diameter (HR 0.899, 95% CI 0.816–0.991, *p* = 0.032) as independent predictors of atrial arrhythmia recurrence (Table 3).

**Table 3:** Predictors of AF/AFL/AT recurrence.

	Univariate	Multivariate				
Variable	HR	95% CI	*p* value	HR	95% CI	*p* value
Age	0.987	0.937–1.040	0.627			
Men	3.019	0.402–22.69	0.283			
BMI	0.942	0.822–1.079	0.390			
Duration of AF	1.010	0.988–1.032	0.391			
Long‐standing persistent AF	0.279	0.659–4.247	1.672			
LA diameter	0.911	0.833–0.996	0.040	0.899	0.816–0.991	0.032
LVEF	1.010	0.949–1.076	0.748			
BNP	0.996	0.988–1.004	0.316			
eGFR	1.025	0.990–1.060	0.162			
High‐DF sites in LA, n	1.045	0.947–1.153	0.379			
High‐DF sites in RA, n	0.999	0.893–1.119	0.992			
LVA/LA surface area after PVI	0.993	0.895–1.102	0.894			
LVA/RA surface area after PVI	1.030	1.003–1.057	0.028	1.031	1.005–1.058	0.018

Abbreviations: AF = atrial fibrillation, AFL = atrial flutter, AT = atrial tachycardia, BNP = B‐type Natriuretic Peptide, BMI = body mass index, CI = confidence interval, DF = dominant frequency; GFR = glomerular filtration rate, HR = hazard ratio, LA = left atrium, LVA = low voltage areas, LVEF = left ventricular ejection fraction, PVI = pulmonary vein isolation, RA = right atrium.

*Note:* Due to the limited number of events, the multivariable analysis should be interpreted with caution.

## 4 Discussion

### 4.1 Major Findings

In this multicenter randomized trial, adjunctive DF‐guided ablation following PVI did not result in a statistically significant improvement in arrhythmia‐free survival compared with PVI alone. Importantly, this finding should not be regarded as a failure but rather as an indication of the complex and heterogeneous nature of the atrial substrate in persistent AF. The study provides novel mechanistic and clinical insights. Specifically, DF mapping was feasible and safe, and the extent of right atrial low‐voltage areas (LVA) emerged as an independent predictor of recurrence. Accordingly, the present findings should be interpreted as hypothesis‐generating rather than definitive evidence regarding the efficacy of DF‐guided ablation.

### 4.2 Interpretation of Findings

The hypothesis underlying DF‐guided ablation stems from the assumption that regions harboring the highest activation frequencies may act as AF drivers or rotors [15, 16, 17, 18]. Prior studies have reported variable success in targeting high DF sites, particularly in patients with persistent AF, as demonstrated in both experimental and clinical settings [11, 12, 13, 14, 16, 17, 18]. In addition, previous randomized studies targeting putative AF drivers have also yielded inconsistent results, further highlighting the complexity and heterogeneity of substrate‐based ablation strategies in persistent AF [19]. However, in the PAD‐AF trial, we found no significant benefit of adjunctive DF ablation, despite rigorous procedural standardization and endpoint adjudication. Notably, AF termination to sinus rhythm was not observed in any patient during DF‐guided ablation. If high dominant‐frequency sites represented true localized AF drivers or rotors, some degree of acute AF termination or at least cycle length prolongation might be expected during ablation. The absence of such responses suggests that high‐DF sites identified using the present methodology may reflect regions of rapid activation rather than critical sustaining sources. These results suggest that DF sites, as currently identified using spectral analysis during ongoing AF, may not consistently represent critical driver regions. Instead, they may reflect passive bystander activity or be affected by local tissue anisotropy, wavebreaks, or fractionated conduction. Moreover, the spatial and temporal instability of high DF sites could limit their utility as fixed ablation targets [20].

### 4.3 Clinical Implications

Although adjunctive DF ablation did not achieve statistical superiority, the numerical trend toward improved outcomes (81.3% vs 68.9%) suggests that DF analysis may contribute to individualized substrate evaluation in selected patients. Importantly, the absence of high‐DF sites was associated with the most favorable prognosis, suggesting that DF analysis may have value as a prognostic marker rather than as a universal ablation target. This observation highlights that not all patients with persistent AF harbor frequency‐defined driver regions, and that the lack of such sites may identify a subset of patients with more favorable substrate characteristics.

### 4.4 Mechanistic Insights Into Atrial Substrate

Our findings demonstrated a limited spatial overlap between high‐DF sites and LVA, highlighting a dissociation between functional activation patterns and structural remodeling. In addition, AF termination was not observed during DF‐guided ablation, further suggesting that high‐DF sites identified using the present methodology may not represent critical AF‐maintaining sources. Taken together, these observations indicate that high‐DF regions may reflect areas of rapid activation influenced by local conduction heterogeneity, anisotropy, or wavebreaks, rather than stable drivers [20]. The spatial and temporal variability of DF sites further limits their utility as fixed ablation targets. These findings underscore the complex and heterogeneous nature of the atrial substrate in persistent AF and support the concept that functional and structural remodeling represent distinct but interacting components of AF maintenance, emphasizing the need for integrative substrate‐based strategies beyond frequency‐guided ablation alone.

### 4.5 Low‐Voltage Areas and Recurrence Risk

Among all substrate metrics, the extent of RA LVA emerged as an independent predictor of AF recurrence. This aligns with growing evidence linking atrial fibrosis with arrhythmia persistence and recurrence [21, 22, 23]. Structural remodeling may underlie both the failure of PVI and the limited efficacy of functional mapping strategies like DF analysis. Interestingly, LA diameter was inversely associated with recurrence in the present analysis. This counterintuitive finding should be interpreted with caution, given the limited sample size, potential collinearity with other substrate variables, and the exploratory nature of the multivariable model. Our results reinforce the concept that substrate modification in persistent AF should be guided by integrated evaluation of structural and functional characteristics, and that low‐voltage mapping, particularly in the RA, warrants greater attention in ablation planning [24].

### 4.6 Procedural Safety and Technical Feasibility

This study also confirmed the feasibility and safety of DF mapping and ablation as an adjunct to PVI. No major procedure‐related complications including stroke, tamponade, pulmonary vein stenosis, or esophageal injury were observed. This suggests that DF‐guided strategies can be safely implemented in experienced centers, potentially enhancing procedural tailoring without compromising safety [19].

### 4.7 Future Directions

In the current era of pulsed field ablation (PFA), the role of RF‐based substrate modification may appear limited. However, frequency‐domain analysis remains applicable across energy sources and may provide added value when combined with advanced mapping or artificial intelligence‐assisted localization [25]. Larger‐scale trials integrating DF analysis with modern ablation technologies are warranted.

### 4.8 Limitations

Several limitations must be acknowledged. First, the number of randomized patients was modest, which limited the statistical power to detect small but potentially clinically meaningful differences. Patient enrollment was also impacted by the COVID‐19 pandemic, further restricting trial size. In addition, because the number of outcome events was relatively limited, the multivariable Cox regression analysis should be interpreted with caution. Second, the relatively strict exclusion criteria were selected to reduce heterogeneity in atrial structural remodeling and facilitate mechanistic interpretation of DF mapping. However, these criteria may limit the generalizability of the findings to broader persistent AF populations. Third, the trial was conducted using radiofrequency energy, whereas PFA is rapidly becoming the predominant modality. Although this limits direct applicability to the current era, frequency‐domain analysis remains relevant across energy sources and may retain value when integrated with modern mapping and ablation technologies. Fourth, DF analysis was performed during ongoing AF, which may have contributed to temporal and spatial instability of the signals and limited identification of consistent driver sites. In addition, formal repeated‐measure assessment of temporal stability and inter‐observer reproducibility of DF identification was not prospectively performed, which may have affected reproducibility of the mapping results. Finally, although the study was conducted across multiple centers with standardized protocols, differences in mapping density and operator experience could not be fully eliminated. Because not all patients undergoing catheter ablation for non‐paroxysmal atrial fibrillation during the study period were formally screened for study participation, selection bias cannot be excluded. Therefore, the study population may not fully represent the overall population of patients undergoing ablation for persistent AF, which may limit the generalizability of the present findings. However, the multicenter design and the consecutive enrollment of eligible patients at each participating center may partially mitigate this concern, although this limitation should be considered when interpreting the results.

## 5 Conclusions

In this multicenter randomized trial of patients with persistent AF, adjunctive DF‐guided ablation following PVI did not result in a statistically significant improvement in arrhythmia‐free survival compared with PVI alone. However, the absence of high‐DF sites was associated with favorable outcomes, suggesting a potential role of DF mapping in patient stratification. In addition, right atrial low‐voltage burden emerged as an independent predictor of recurrence, underscoring the importance of structural remodeling in ablation outcomes.

Taken together, these findings suggest that while DF‐guided ablation may not provide consistent incremental benefit as a standalone strategy, DF analysis remains valuable for mechanistic insight and substrate characterization. The feasibility and safety of DF mapping support its potential integration with emerging ablation technologies and individualized ablation strategies. Further studies are warranted to refine the role of frequency‐domain mapping in the evolving landscape of AF ablation.

## Conflicts of Interest

The authors declare no conflicts of interest.

## Data Availability

The data that support the findings of this study are available on request from the corresponding author. The data are not publicly available due to privacy or ethical restrictions.
